# Characterization of Deltacoronavirus in Black-Headed Gulls (*Chroicocephalus ridibundus*) in South China Indicating Frequent Interspecies Transmission of the Virus in Birds

**DOI:** 10.3389/fmicb.2022.895741

**Published:** 2022-05-12

**Authors:** Kan-Kan Chu, Zhi-Jian Zhou, Qiong Wang, Sheng-Bao Ye, Ling Guo, Ye Qiu, Yun-Zhi Zhang, Xing-Yi Ge

**Affiliations:** ^1^College of Biology & Hunan Provincial Key Laboratory of Medical Virology, Hunan University, Changsha, China; ^2^Yunnan Province Key Laboratory of Anti-pathogenic Plant Resources Screening (Cultivation), Yunnan Province Key University Laboratory of Zoonoses Cross-Border Prevention and Quarantine, Institute of Preventive Medicine, School of Public Health, Dali University, Dali, China

**Keywords:** deltacoronavirus, black-headed gull, phylogeny, interspecies transmission, codivergence

## Abstract

Deltacoronavirus (DCoV) is a genus of coronavirus (CoV) commonly found in avian and swine, but some DCoVs are capable of infecting humans, which causes the concern about interspecies transmission of DCoVs. Thus, monitoring the existence of DCoVs in animals near communities is of great importance for epidemic prevention. Black-headed gulls (*Chroicocephalus ridibundus*) are common migratory birds inhabiting in most urban and rural wetlands of Yunnan Province, China, which is a typical habitat for black-headed gulls to overwinter. Whether Yunnan black-headed gulls carry CoV has never been determined. In this study, we identified three strains of DCoVs in fecal samples of Yunnan black-headed gulls by reverse-transcriptional PCR and sequenced their whole genomes. Genomic analysis revealed that these three strains shared genomic identity of more than 99%, thus named DCoV HNU4-1, HNU4-2, and HNU4-3; their NSP12 showed high similarity of amino acid sequence to the homologs of falcon coronavirus UAE-HKU27 (HKU27), houbara coronavirus UAE-HKU28 (HKU28), and pigeon coronavirus UAE-HKU29 (HKU29). Since both HKU28 and HKU29 were found in Dubai, there might be cross-border transmission of these avian DCoVs through specific routes. Further coevolutionary analysis supported this speculation that HNU4 (or its ancestors) in black-headed gulls originated from HKU28 (or its homologous strain) in houbara, which was interspecies transmission between two different avian orders. In addition, interspecies transmission of DCoV, from houbara to falcon, pigeon and white-eye, from sparrow to common-magpie, and quail and mammal including porcine and Asian leopard cat, from munia to magpie-robin, was predicted. This is the first report of black-headed gull DCoV in Asia which was highly homolog to other avian DCoVs, and the very “active” host-switching events in DCoV were predicted, which provides important reference for the study of spread and transmission of DCoVs.

## Introduction

Coronavirus (CoV) usually refers to the viruses of the sub-family of *Orthocoronavirinae* in the family of *Coronaviridae*. In the recent two decades, CoVs have caused three intercontinental epidemics, including the ongoing worldwide pandemic of COVID-19 with more than 500 million infections and almost 6 million deaths. It has been reported that human epidemic CoVs were originated from zoonotic CoVs which were reserved in wild animals and infected humans *via* occasional interspecies transmission (Ge et al., 2013; Corman et al., 2015; Sabir et al., 2016; Tao et al., 2017; Cui et al., 2019). A variety of animals can serve as the reservoir of CoVs, including bats, birds, mice, and even domestic animals (Chen et al., 2020; Zhou et al., 2021). So far, more than 40 species of CoVs have been reported all over the world by the International Committee on Taxonomy of Viruses (ICTV) (Zhou et al., 2021). The *Coronaviridae* Study Group (CSG) of ICTV classified coronaviruses into four genera: *Alphacoronavirus*, *Betacoronavirus*, *Gammacoronavirus*, and *Deltacoronavirus*, by analyzing five essential protein domains (3CL-pro, NiRAN, RdRp, ZBD, and HEL1) combined with phylogeny and genomic structure (Coronaviridae Study Group of the International Committee on Taxonomy of Viruses et al., 2020). Alphacoronavirus and betacoronavirus mainly infect mammals (Hamre and Procknow, 1966; McIntosh et al., 1967; van der Hoek et al., 2004; Vlasova et al., 2011; Lin et al., 2017; Saldanha et al., 2019). Gammacoronavirus and deltacoronavirus (DCoV) mainly infect birds (Mihindukulasuriya et al., 2008; Woo et al., 2009, 2012; Hepojoki et al., 2017).

Although birds are major hosts for DCoV, this virus also infects swine, indicating potential interspecies transmission from birds to mammals which is supported by the evolutionary relationship between sparrow DCoV and porcine DCoV (PDCoV) (Woo et al., 2012; Chen et al., 2018). Notably, PDCoV was reported to infect humans (Lednicky et al., 2021). Since birds and swine are common animals inhabiting in or near human communities, some birds are frequently migrating for a long distance, leading to a concern about potential epidemics caused by DCoV. Therefore, it is important to monitor DCoV in these host animals.

Black-headed gull (*Chroicocephalus ridibundus*) is a migratory bird widely distributed in Eurasia and on the East Coast of North America (Ushine et al., 2017). Xinjiang, Inner Mongolia, and Heilongjiang in China are their major breeding grounds. In winter, flocks of black-headed gulls migrate from Siberia to Southern China to overwinter, and Yunnan province is a typical habitat for black-headed gulls during winters. Since the 1980s, every year from November to March, tens of thousands of black-headed gulls have been inhabiting in Yunnan where they closely contact with local animals, raising the concern about epidemic viruses such as CoV transmitted from black-headed gulls to others (Liao et al., 2019). According to previous studies, gammacoronavirus has been detected in gulls (Domanska-Blicharz et al., 2014; Wille et al., 2016). As far as we know, only a 461 bp fragment in *rdrp* gene of black-headed gull DCoV (BHG-DCoV) has been reported in Finland (Hepojoki et al., 2017). However, there is no study on the whole genome of black-headed gull CoV.

In this study, we collected the fecal samples of black-headed gulls in Kunming and Dali, two major cities in Yunnan, for DCoV detection. As a result, three strains of BHG-DCoV strains (HNU4-1, HNU4-2, and HNU4-3) were identified with whole-genome identity of more than 99%. Further genome analysis revealed that the genome of HNU4 showed high identity of more than 96% to those of HKU27, HKU28, and HKU29 found in United Arab Emirates, indicating a potential transboundary transmission. The differentiation time analysis and coevolution analysis support that HNU4 (or its ancestors) in black-headed gull (from *Charadriiformes*) may spread from HKU28 (or its homologous strain) in houbara (from *Gruiformesor*). According to the sequence of the five essential domains for CoV taxonomy, HNU4 could be classified into the same species of *White-eye CoV-HKU16*, and further analysis showed that *White-eye CoV-HKU16* like coronavirus had been found in six orders of birds, suggesting the activity of host jumping ability of this coronavirus. Our finding reveals the widespread of DCoV as well as its potential transboundary and interspecies transmission. Therefore, more attention should be paid on DCoV in wide birds to prevent potential epidemics.

## Materials and Methods

### Sample Collection

From January to March 2021, 460 feces samples of black-headed gulls were collected at seven sampling points near Erhai Lake in Dali, Yunnan Province, and Dianchi Lake in Kunming, Yunnan Province. All samples were placed in virus transport medium (VTM) and kept in dry ice for transportation to the laboratory and stored at −80°C until use.

### RNA Extraction and Coronavirus Reverse-Transcriptional PCR Screening

Viral RNA was extracted from the fecal samples using MagaBio plus Virus DNA/RNA Purification Kit II (BioFlux, China) following the manufacturer’s instructions. RNA extracted from fecal was washed off with 70-μl RNase-free water and stored in −80°C as a template for reverse-transcriptional PCR (RT-PCR). Initial CoV screening was performed by amplifying a 440-bp fragment of the *rdrp* gene of CoVs using primers DCoV-F (5′-GTGGVTGTMTTAATGCACAGTC-3′) and -R (5′-TACTGYCTGTTRGTCATRGTG-3′) (Lau et al., 2018). Primescript™ one-step RT-PCR kit ver. 2 (Takara) was used for PCR amplification. About 20 μl PCR mixture included 0.8 μl Primescript 1 step enzyme, 10 μl 2 × 1 step buffer, 5.2 μl ddH_2_O, and 2 μl extracted RNA. The mixtures were amplified for 40 cycles as 94°C for 30 s, 48°C for 30s, and 72°C for 40 s and a final extension at 72°C for 10 min in an automated thermal cycler (Applied Biosystems). To confirm the bird species, the mitochondrial cytochrome b (*cytb*) gene was amplified and sequenced of the samples.

The PCR products were gel-purified and sequenced with an ABI Prism 3,700 DNA analyzer (Applied Biosystems), using the PCR primers. The sequences of the PCR products were compared with known sequences of the *rdrp* genes of CoVs in the GenBank database.

### Complete Genome Sequencing

Three complete genomes of BHG-DCoVs were amplified and sequenced using RNA extracted from black-headed gull feces as templates. RNA was amplified with degenerate primers, which were designed by multiple alignments of other coronavirus genomes with the complete genome, using Primescript one-step RT-PCR kit version 2. Additional primers were designed according to the results of the first and subsequent rounds of sequencing. The 5′ and 3′ genome end sequences were obtained by 5′ and 3′ race (Roche), respectively. The expected size of PCR products was purified by gel and directly sequenced. The sequence was assembled to obtain the full-length genome sequence.

### Genomic and Phylogenetic Analysis

Multiple sequence alignment was performed by MAFFT v7.149 in BioAider v1.334 (Katoh and Standley, 2013; Zhou et al., 2020). The maximum likelihood phylogenetic tree of CoVs’ RdRp was constructed by IQ-tree v1.6.10 in PhyloSuite program with 10,000 ultrafast bootstraps, and the most appropriate substitution model of aa was calculated using ModelFinder according to the Bayesian information criterion (BIC) method (Nguyen et al., 2015; Zhang et al., 2019). The phylogenetic tree of ORF1ab, NIRAN, 3CL-pro, HEL1, and S protein was performed using MEGA7 with 1,000 bootstraps in neighbor-joining method (Kumar et al., 2016).

### Estimation of Divergence Dates

The *rdrp* gene was aligned using MAFFT program with codon method in BioAider v1.334. Then, we detected the temporal structure in these *rdrp* gene sequences by TreeTime program (Sagulenko et al., 2018). The correlation coefficient of R^2^ was 0.01, and there was very weak signal between sampling time and genetic distance in the data (Supplementary Figure 1). Therefore, we used a uniform distribution priori value (from 8 × 10^–5^ to 2 × 10^–4^ subs/site/year) according to the latest report of evolution rate of *rdrp* gene in *DCoV* (Wang et al., 2021). Then, we ran a Markov chain of 10 million steps with sampling every 1,000 steps in BEAST v1.10.4 (Drummond et al., 2012). The mean evolution rate and time of the most recent common ancestor (tMRCA) were calculated under uncorrelated lognormal relaxed clock. The most appropriate substitute model of nucleotide was GTR + F + G4 calculated by ModelFinder according to BIC method. We checked the effective sample size (ESS) of parameters in Tracer v1.7 program and ensured that they all reached convergence (ESS > 200). Finally, the maximum clade credibility (MCC) tree was obtained by discarding the first 10% of states in Tree Annotator package.

### Coevolutionary Analyses of Virus and Host

Paired evolutionary trees of DCoVs and their corresponding hosts were used to examine the evolutionary relationship. Some DCoV strains without host records were excluded in this analysis. In addition to our samples, only one or two strains (from different countries) were reserved as the representative for these highly similar sequences with the same host. The host evolutionary tree and topologies were obtained from the TimeTree website^1^ by entering the Latin text of all species to be analyzed (Kumar et al., 2017). Then, we constructed the viral phylogenetic tree based on the *rdrp* gene using Mrbayes v3.2.6 program (Ronquist et al., 2012). We run three hot and one cold Markov Chain Monte Carlo (MCMC) chains in Mrbayes, and the appropriate substitution and site-variation model of nucleotides was set to GTR + F + I + G4 which was tested and estimated by ModelFinder software according to BIC method. We performed two independent runs with total generations of 2,000,000 for each run and sampled every 100 generations. The first 10% burned samples were discarded, and the 50% majority rule tree was generated using the remaining samples. We checked the ESS value of each parameter in Tracer v1.6 program and ensured that they all reach convergence (ESS > 200).

According to previous research on the evolutionary history of RNA virus (Shi et al., 2018), we examined the co-divergence (coevolution) and cross-host transfer events among DCoVs and their hosts using eMPRess program (Santichaivekin et al., 2021). The consistency test of paired host–virus tree in eMPRess was conducted based on the duplication-transfer-loss (DTL) model, using a maximum parsimony approach (MPR) to find a “best” mapping of the virus tree onto the host tree which minimized the total “cost.” The null hypothesis is that the reconciliation score of the original dataset was due to change in eMPRess. The “cost” of every event was set as follows: co-divergence = 0 (fixed), duplication = 1, transfer (aka host switch) = 1.2, and loss = 1. The statistical test of co-divergence was conducted by comparing the estimated cost to null distributions calculated from 100 random permutations of the host tip mappings.

## Results

### Detection Results of Black-Headed Gull Coronavirus

**FIGURE 1:**
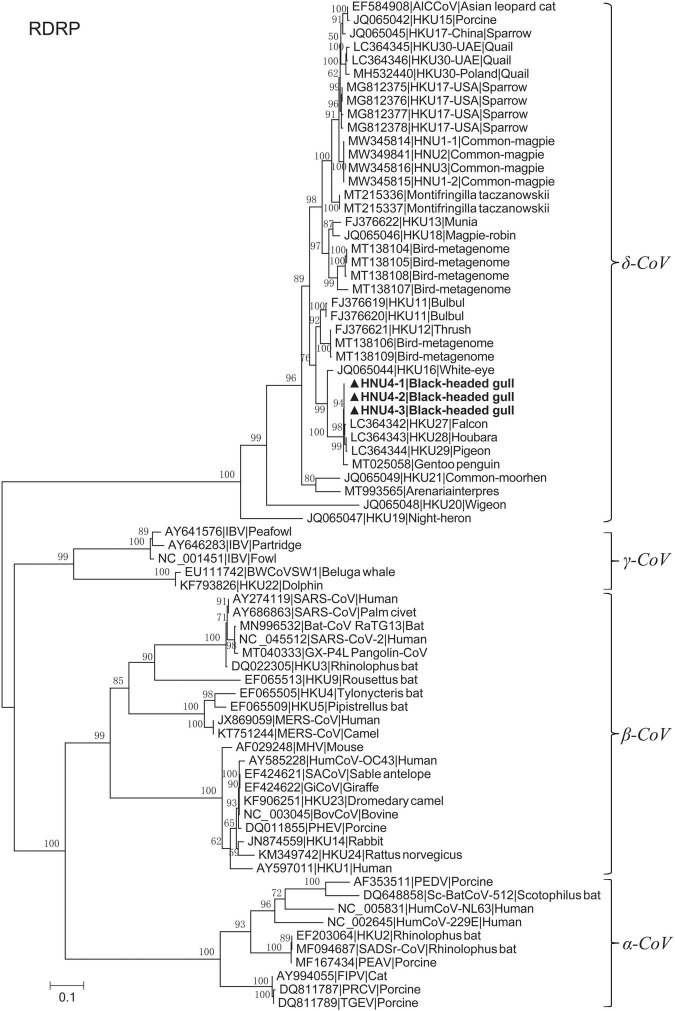
Maximum likelihood tree of RdRp fragment with LG + I + G4 substitution model based on amino acids. The bold ones are BHG-DCoV-HNU4-1, BHG-DCoV-HNU4-2, and BHG-DCoV-HNU4-3 found in this study.

A total of 460 fecal samples were collected. Reverse-transcriptional PCR detection showed that three samples were positive of DCoV, with a total positive rate of 0.65%. These positive samples were collected in Cuihu Park, Kunming (Table 1). The three PCR products were sequenced, and based on the sequences, we constructed a phylogenetic tree together with other coronaviruses, preliminarily confirming that the sequences we detected could be clustered into the genus of *deltacoronavirus* (Figure 1). Especially, the RdRp sequences were highly similar to those of DCoVs HKU27, HKU28, and HKU29 detected in Dubai in 2018 (Lau et al., 2018), with identities of 99.290–99.645%.

**TABLE 1:** Detection results of DCoV in fecal samples of lack-headed gull in Yunnan Province.

Sampling point	Samples	Positive samples	Infection rate (%)
A	63	0	0
B	13	0	0
C	112	0	0
D	139	3	2.16
E	51	0	0
F	51	0	0
G	31	0	0
Total	460	3	0.65

*A, Erhai moon Wetland Park, Dali; B, Xingsheng Bridge, Dali; C, Xiaoputuo, Wase Town, Dali; D, Cuihu Park, Kunming; E, Haigeng Park, Kunming; F, Haigeng dam, Kunming, G, Daguan Park, Kunming.*

### Genome Analysis of the Black-Headed Gull Deltacoronavirus

**FIGURE 2:**
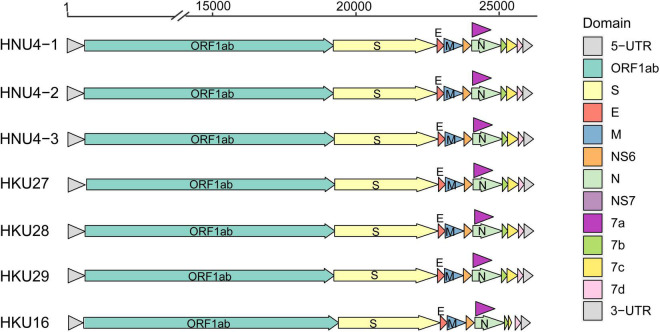
Genomic structure of 7 DCoV strains.

In order to further reveal the genetic and evolutionary characteristics of the potential DCoVs detected in black-headed gulls, we amplified and sequenced the whole genome of the three DCoVs positives. As a result, three whole-genome sequences of 26,159 nt were obtained. Then, we annotated the genomes by referring to HKU29 genome (LC364344.1) and identified 10 typical open reading frames (ORFs) of coronavirus which were aligned as 5′ untranslated region (5′ UTR)-replicase ORF1ab-spike (S)-envelope (E)-membrane (M)-Nucleocapsid (N)-3′ UTR. Moreover, additional ORFs coding non-structural (NS) proteins (NS6, NS7a, NS7b, NS7c, and NS7d) were identified (Figure 2). The identities among the three genomes ranged from 99.382 to 99.957%, and they showed similarity of 96.606–96.911% to DCoVs HKU27, HKU28, and HKU29 (Table 2). Hence, we named the three DCoV strains in black-headed gulls as BHG-DCoV HNU4-1, HNU4-2, and HNU4-3.

**TABLE 2:** Comparison of genomic features and identities of eight DCoV strains.

			Nucleotide/amino acid identity (%)																							
Corona virus	Genome size (base)	G + C content	BHG-DCoV-HNU4-1								BHG-DCoV-HNU4-2								BHG-DCoV-HNU4-3							
			Genome	3CL-pro	NIRAN	RdRp	ZBD	HEL1	S	N	Genome	3CL-pro	NIRAN	RdRp	ZBD	HEL1	S	N	Genome	3CL-pro	NIRAN	RdRp	ZBD	HEL1	S	N
WECoV-HKU16	26041	0.40	73.30/ –	77.42/ 86.97	79.01/ 85.88	86.26/ 95.38	84.91/ 97.90	87.22/ 96.80	52.06/ 46.23	80.50/ 83.29	73.30/ –	77.42/ 86.97	79.01/ 85.88	86.26/ 95.38	84.91/ 97.90	87.22/ 96.80	52.01/ 46.23	80.50/ 83.29	73.30/ –	77.42/ 86.97	78.88/ 85.47	86.44/ 95.38	84.21/ 97.90	87.50/ 96.80	52.03/ 46.31	80.31/ 83.28
FalCoV UAE-HKU27	26155	0.39	96.61/ –	96.63/ 99.02	97.46/ 97.71	97.16/ 99.65	97.90/ 100.00	96.91/ 99.54	92.59/ 93.93	97.19/ 97.68	96.61/ –	96.63/ 99.02	97.46/ 97.71	97.16/ 99.65	97.90/ 100.00	96.91/ 99.54	92.51/ 93.93	97.19/ 97.68	96.60/ –	96.63/ 99.02	97.33/ 97.33	97.51/ 99.65	97.19/ 100.00	97.09/ 99.54	92.59/ 94.02	97.19/ 97.68
HouCoV UAE-HKU28	26155	0.39	96.93/ –	96.53/ 99.02	98.22/ 98.47	97.04/ 99.47	99.30/ 100.00	97.09/ 99.54	93.89/ 95.77	97.87/ 98.26	96.93/ –	96.53/ 99.02	98.22/ 98.47	97.04/ 99.47	99.30/ 100.00	97.09/ 99.54	93.82/ 95.77	97.87/ 98.26	96.88/ –	96.53/ 99.02	98.09/ 98.09	97.51/ 99.47	98.60/ 100.00	97.44/ 99.54	93.84/ 95.68	97.67/ 98.26
PiCoV UAE-HKU29	26162	0.39	96.93/ –	96.74/ 99.67	98.09/ 98.09	96.92/ 99.29	99.30/ 100.00	97.09/ 99.54	93.87/ 95.60	97.87/ 98.26	96.93/ –	96.74/ 99.67	98.09/ 98.09	96.92/ 99.29	99.30/ 100.00	97.09/ 99.54	93.79/ 95.60	97.87/ 98.26	96.88/ –	96.74/ 99.67	97.96/ 97.71	97.40/ 99.29	98.60/ 100.00	97.44/ 99.54	93.81/ 95.52	97.67/ 98.26
GPCoV*	–	–	–	90.99/ 97.720	94.28/ 96.18	95.20/ 98.93	95.09/ 97.90	96.19/ 99.09	–	–	–	90.99/ 97.72	94.28/ 96.183	95.20/ 98.93	95.09/ 97.90	96.19/ 99.09	–	–	–	90.99/ 97.720	94.15/ 95.80	95.26/ 98.93	93.69/ 97.90	96.91/ 99.09	–	–
BHG-DCoV-HNU4-1	26159	0.398	–	–	–	–	–	–	–	–	99.99/ –	100.00/ 100.00	100.00/ 100.00	100.00/ 100.00	100.00/ 100.00	100.00/ 100.00	99.92/ 100.00	100.00/ 100.00	99.40/ –	100.00/ 100.00	99.87/ 99.62	98.64/ 100.00	98.60/ 100.00	98.24/ 100.00	99.81/ 99.58	99.81/ 100.00
BHG-DCoV-HNU4-2	26159	0.398	99.99/ –	100.00/ 100.00	100.00/ 100.00	100.00/ 100.00	100.00/ 100.00	100.00/ 100.00	99.92/ 100	100.00/ 100.00	–	–	–	–	–	–	–	–	99.40/ –	100.00/ 00100	99.87/ 99.62	98.64/ 100.00	98.60/ 100.00	98.24/ 100.00	99.72/ 99.58	99.81/ 100.00
BHG-DCoV-HNU4-3	26159	0.398	99.40/ –	100.00/ 100.00	99.87/ 99.62	98.64/ 100.00	98.60/ 100.00	98.24/ 100.00	99.81/ 99.58	99.81/ 100.00	99.40/ –	100.00/ 100.00	99.87/ 99.62	98.64/ 100.00	98.60/ 100.00	98.24/ 100.00	99.72/ 99.58	99.81/ 100.00	–	–	–	–	–	–	–	–

*Gentoo Penguin virus (GPCoV) is a deltacoronavirus from Antarctic penguins. It currently has only ORF1ab fragment, and the GenBank accession number is MT025058.1.

The 3CL-pro, NiRAN, RdRp, ZBD, and HEL1 are the hallmark domains for classification of coronaviruses (International Committee on Taxonomy of Viruses Executive Committee et al., 2020). In order to confirm the taxonomic status of HNU4-1, HNU4-2, and HNU4-3, we compared the aa sequences of these domains with the known DCoVs *White-eye coronavirus HKU16* (WECoV-HKU16), HKU27, HKU28, and HKU29. The result showed that all the eight viruses could be classified into the same species under the genus of deltacoronavirus (Table 2). Although the eight DCoVs were classified into the same species, the structural proteins of the BHG-DCoV, HKU27, HKU28, and HKU29 shared high similarity while WECoV-HKU16’s were quite distinct from the others (Table 2).

### Amino Acid Variants in ORF1ab and Other Main Proteins

**FIGURE 3:**
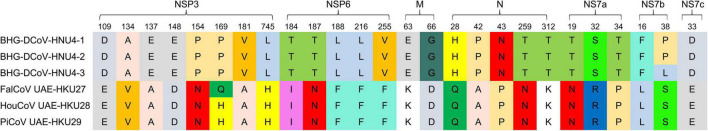
Amino acid variations on NSP3, NSP6, M, N, NS7a, NS7b, and NS7c proteins of six DCoV strains.

In order to further compare the differences among BHG-DCoV and HKU27, HKU28, and HKU29, we compared the amino acids encoded in their coding regions. The results showed that there were amino acid changes in ORF1ab. We used the cleavage sites of 16 NSPs of ORF1ab analyzed previously to predict the cleavage sites of ORF1ab of BHG-DCoV (Lau et al., 2018). The results showed that the mutations of ORF1ab mainly occurred in NSP3 and NSP6 (Figure 3). BHG-DCoV and HKU27, HKU28, and HKU29 also had different degrees of amino acid mutations in M, N, NS7a, NS7b, and NS7c. Among them, the most amino acid mutation was N protein, with a total of five amino acids mutated, and the least was NS7c, with only one amino acid mutation (Figure 3).

### Amino Acid Variants in Spike Protein

**FIGURE 4:**
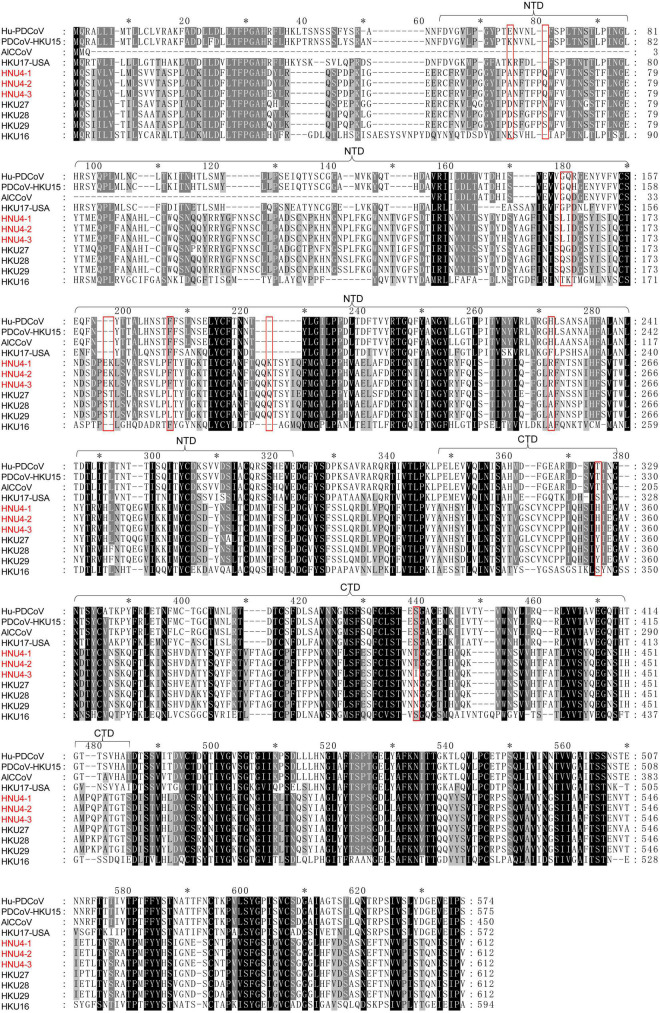
Multiple comparison of S1 fragment among BHG-DCoVs, HKU27, HKU28, HKU29, HKU16, Hu-PDCoV (MW685622.1), PDCoV-HKU15 (JQ065042.2), HKU17-USA (MG812377.1), and AlCCoV (EF584908.1). The annotation of NTD and CTD domains in S1 was with reference to PDCoV (Shang et al., 2018). The red boxes indicate unique sites of BHG-DCoVs compared to HKU27, HKU28, and HKU29.

The difference of S protein among BHG-DCoV, HKU27, HKU28, HKU29, mammalian DCoV, and sparrow coronavirus HKU17-USA (close to PDCoV) was compared (Figure 4 and Supplementary Figure 2). The S protein was quite different between avian DCoV (except HKU17-USA) and mammalian DCoV, and the BHG-DCoV only shared about 45% aa identity with mammalian DCoV (Supplementary Table 1). Besides, there were multiple deletions/insertions and mutations in the NTD domain of S1 region, which may be a possible region for host receptor utilization restriction (Figure 4). However, the difference in the S2 region was relatively small (Supplementary Figure 2). We further analyzed the variation in BHG-DCoV compared with HKU27, HKU28, and HKU29 in S1 region. The result showed that there were nine sites with completely different amino acids between BHG-DCoV, HKU27, HKU28, and HKU29 in S1-NTD (D59A, N/S65Q, Q162L, G/S163I, S179E, T180K, L190F, Q207K, and A253R), and two sites in S1-CTD (Y355H and N419T). Besides, HKU28 was the closest BHG-DCoV on S protein and showed about 95.7% aa identity.

**FIGURE 5:**
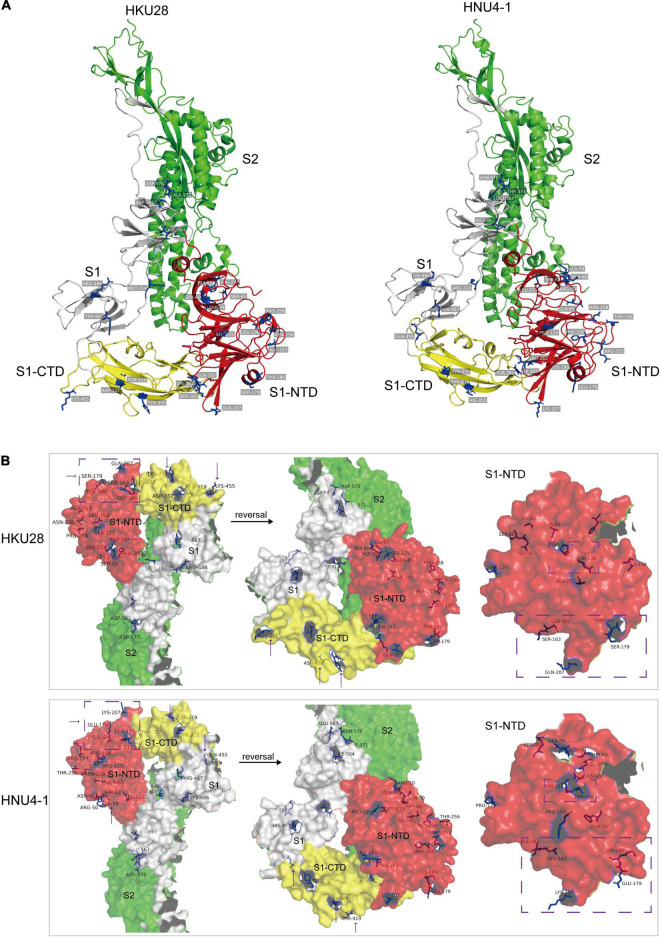
Tertiary structure (chain A) of S protein of HKU28 and HNU4-1. These structures were predicted by SWISS-MODEL server (https://swissmodel.expasy.org/interactive) using S protein of PDCoV as template (PDB ID: 6bfu.1). **(A)** Different colors indicate different regions of the S protein, red (S1-NTD), yellow (S1-CTD), gray (fragment in S1), and green (S2). **(B)** Surface of the S protein, differential residue sites between HKU28 and HNU4-1 were marked in blue.

To assess the impact of mutations on S protein structure and function, we compared the differences in primary and tertiary structures of HNU4-1 and HKU28. In primary sequence, there were a total of 50 aa mutations and one aa insertion in S protein of HNU4-1 compared to HKU28 (Supplementary Table 2). Of these, 16 mutations were in S1-NTD and five mutations were in S1-CTD. We subsequently predicted the tertiary structures of S protein of HNU4-1 and HKU28 by homology modeling method, respectively (Figure 5). The tertiary structure of HKU28 covers the region from aa 51 to 1061, and the tertiary structure of HNU4-1 covers the region from aa 46 to 1062, the chain A as shown in Figure 5A. In general, the tertiary structure of the S protein of HNU4-1 and HKU28 was very similar. However, some mutation sites caused significant changes in residues conception, such as D59A, Q162L, S163I, S179E, T180K, Q207K, N256T, and P258N in S1-NTD, and Y355H, N419T, and K455Q in S1-CTD (Figure 5B). Notably, Q162L, S163I, S179E, T180K, and Q207K are at the top of the NTD, and Y355H, N419T, and K455Q are also at the top of the CTD; we speculate that it may be related to host receptor utilization. However, avian receptor utilization for DCoV is currently unclear, which greatly limits the in-depth exploration of the impact of mutations on function.

### Phylogenetic Analyses of Black-Headed Gull Deltacoronavirus

**FIGURE 6:**
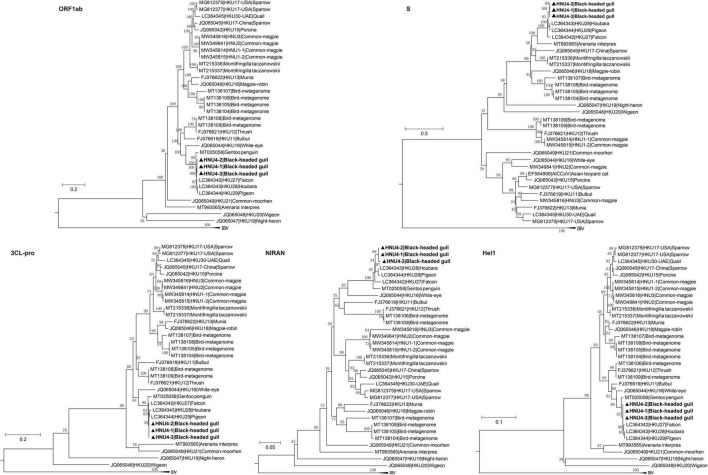
Phylogenetic trees of amino acid sequences of polyprotein 1ab (ORF1ab), spike (S) protein, chymotrypsin-like protease (3CL-pro), superfamily 1 helicase (HEL1), and nidovirus RdRp-associated nucleotidyltransferase (NIRAN). The bold ones are BHG-DCoV-HNU4-1, BHG-DCoV-HNU4-2, and BHG-DCoV-HNU4-3 found in this study.

Based on the amino acid sequences of ORF1ab, S, HEL1, 3CL-pro, and NIRAN, we constructed the phylogenetic trees of BHG-DCoVs and the other 30 DCoVs. The results showed that HNU4-1, HNU4-2, HNU4-3, HKU27, HKU28, and HKU29 were clustered as a sub-branch within a branch including WECoV-HKU16 (Figure 6). Compared with HKU27, HKU28, and HKU29, the three BHG-DCoV are more closely related. It is worth noting that in the phylogenetic tree of ORF1ab, HEL1, 3CL-pro, and NIRAN, they were also clustered with GPCoV found in Antarctica. In the phylogenetic tree constructed by S fragment, except HKU27, HKU28, and HKU29, they also clustered with a *Arenaria interpres* DCoV (ARDCoV) found in Canada (Figure 6; Wille et al., 2021).

### Evolutionary Rate and tMRCA

**FIGURE 7:**
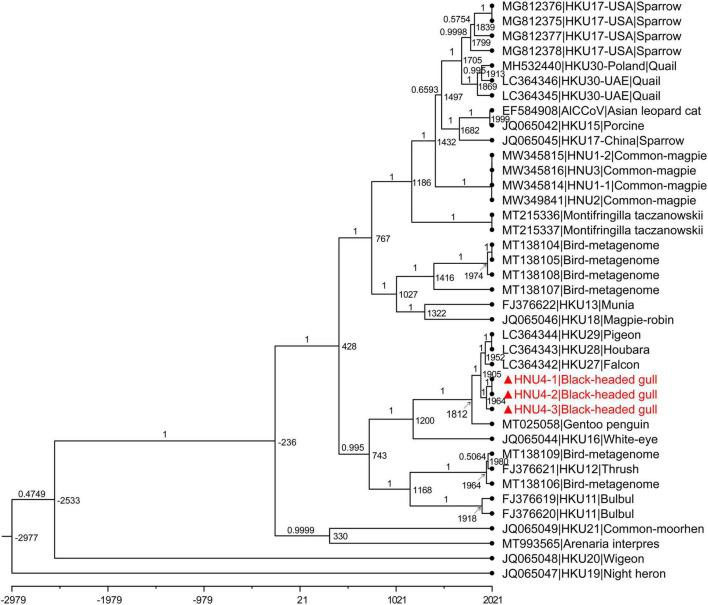
MCC tree with divergence time based on *rdrp* gene. The value near the node indicates the age of the node, and the label on the branch represents the Bayesian posterior probability. The red bold ones are BHG-DCoV-HNU4-1, BHG-DCoV-HNU4-2, and BHG-DCoV-HNU4-3 found in this study.

The estimated mean evolutionary rate of DCoV *rdrp* gene was about 1.49 × 10^–4^ subs/site/year (9.10 × 10^–5^–2.0 × 10^–4^, 95% HPD). The time of the most recent common ancestor (tMRCA) differentiation time of DCoV was about 2977 B.C (6617 B.C–1035 B.C, 95% HPD). The tMRCA of *White-eye coronavirus HKU16* species was about 1200 A.D (594 A.D–1508 A.D, 95% HPD). The tMRCA of HNU4-1, HNU4-2, and HNU4-3 was about 1964 A.D (1914 A.D–1993 A.D, 95% HPD). However, the tMRCA of HKU27, HKU28, and HKU29 was about 1952 A.D (1896 A.D–1983 A.D), and it suggests that they may differentiate earlier than BHG-DCoV (Figure 7).

### Frequent Host-Switching Events of Deltacoronavirus in Birds

**FIGURE 8:**
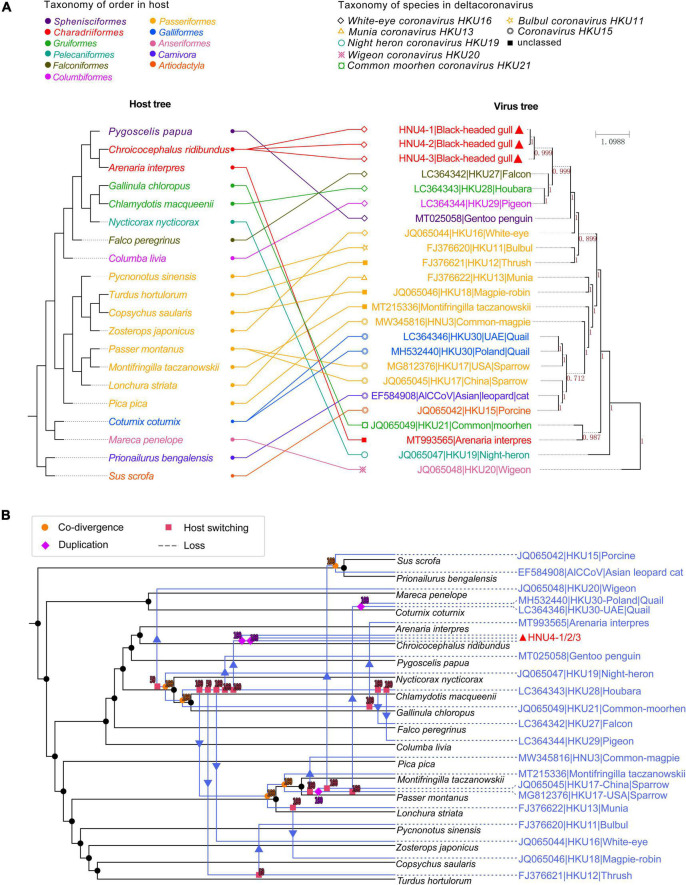
Coevolutionary analysis of virus and host. **(A)** The tanglegram of host and virus tree. The left is the host evolutionary tree, and the same color represents the same order. The right is phylogenetic tree of viral *rdrp* gene, and the same shape represents the same virus species. The value on branch in viral tree represents Bayesian posterior probability. The tanglegram of host–virus was drawn by PhyloSuite software. **(B)** Co-divergence and host switch of DCoV and their hosts. The host tree was marked in black with DCoV tree in blue, and different events were marked with different symbols. Co-divergence, duplication, host switching, and loss events were marked with filled circles, diamond, squares and arrow, and dotted lines, respectively.

The distribution of virus taxa on the host tree showed the host range of DCoV, including 18 species of the Aves class and two species of the Mammalia class (Figure 8 and Supplementary Table 3). The analysis using eMPRess of virus–host mapping detected a total of 23 events, including five co-divergence events, four duplication events, and 14 host-switching events (Figure 8). The global co-divergence test indicated that the co-divergence signal was weak in the host–virus tree, because the “cost” of the original host–virus tree was not significantly less than the randomness (*p*-value > 0.01) (Supplementary Figure 3). Frequent host-switching events indicate that DCoV was actively jumping among different hosts. Especially, during the history of evolution, HKU28 underwent several reliable host-switching events (with supporting values of 100) among which one led to the generation of HNU4 (Figure 8). This indicated that HNU4 might originate from HKU28 *via* cross-species transmission among different avian orders.

Besides, the DCoV in houbara (*Gruiformesor*) looks “restless” and spread to other hosts, such as falcon (*Falco peregrinus*), pigeon (*Columba livia*), and white-eye (*Zosterops japonicus*). Consistent with the previous speculation, the host transfer between houbara and falcons may be caused by the food chain (Lau et al., 2018). It is worth noting that the DCoV in white-rumped snowfinch (*Montifringilla taczanowskii*), sparrow, and munia shared co-divergence with three hosts. Then, the sparrow DCoV spread to common-magpie (*Pica pica*), quail (*Coturnix coturnix*), and mammal (porcine and Asian leopard cat). The munia DCoV may spread to magpie-robin (*Copsychus saularis*). In general, many host-switching events were predicted in DCoV, which imply that DCoV was very “active” in their hosts. However, the current sample of bird DCoV is still limited, and only a single host species has been detected in some orders. Therefore, more convincing evolutionary relationships require more samples to support in future. The large number of potential host-switching events indicates that the prevalence of DCoV in birds is wide and complex, and it is necessary to strengthen monitoring.

## Discussion

Increasing studies have shown that the outbreak of human epidemic is closely related to pathogens reserved in wildlife (Daszak et al., 2000). Especially, the three pathogenic CoVs (SARS-CoV, MERS-CoV, and SARS-CoV-2) causing worldwide epidemics in the recent two decades were all believed to originate from wild animals, revealing a great importance of monitoring CoVs in their natural reservoirs. In this study, we detected three DCoV strains (HNU4-1, HNU4-2, and HNU4-3) in black-headed gulls in Yunnan Province and sequenced their whole genomes. These were the first BHG-DCoVs detected in China and the first whole-genome sequences of BHG-DCoV ever reported. The *rdrp* gene (461bp) identity between BHG-DCoV in Yunnan and BHG-CoV found in Finland is 94.794–95.662%. In view of the high conservation of *rdrp* gene, BHG-DCoV in black-headed gulls may also be diverse. According to the hallmark domain sequences for CoV classification proposed by ICTV, these newly found BHG-DCoV strains could be classified into the species *White-eye coronavirus HKU16*, but the overall genome sequences of the BHG-DCoV showed low identity with *White-eye coronavirus HKU16*, the prototypical virus of the species. In comparison, these BHG-DCoVs genomes were highly similar (>96%) to the genomes of HKU27, HKU28, and HKU29 found in Dubai, indicating the close evolutionary relationship of these avian DCoVs and potential interspecies and transboundary transmission of them.

Despite the high similarity in the whole-genome level, HNU4, HKU27, HKU28, and HKU29 showed a few amino acid variations in several structural and non-structural proteins, including M, N, NSP3, NSP6, NS7a, NS7b, and NS7c. These proteins are critical for CoV life cycle. For instance, NSP3 is a scaffold protein with activity of deubiquitination (DUB) and deISGylation which is necessary for immune evasion (Neuman, 2016; Alfuwaires et al., 2017); NSP6 is capable of activating autophagy which is required for CoV replication (Cottam et al., 2011, 2014; Angelini et al., 2013; Benvenuto et al., 2020); M and N are structural proteins essential for viral assembly (Neuman et al., 2011; McBride et al., 2014; Lee and Lee, 2015). The variance of functional proteins in these closely related DCoVs potentially reveals the adaption of the virus to different hosts, whereas the detailed mechanisms need to be further studied.

Spike protein is the key factor determining the receptor recognition of coronavirus (Gallagher and Buchmeier, 2001; Holmes, 2003). Coronavirus spike has two subunits, S1 and S2. S1 binds to cellular receptor cells for virus attachment, and S2 mediates membrane fusion and virus internalization (Li, 2016). S1 contains two domains related to receptor recognition, S1-NTD binding to polysaccharide and S1-CTD binding to protein (Krempl et al., 1997; Mou et al., 2013; Li, 2016; Shang et al., 2018). In this study, we found that the S protein of BHG-DCoVs, HNU4-1, HNU4-2, and HNU4-3 is highly similar to that of HKU27, HKU28, and HKU29; more detailed studies show that the S1-NTD and S1-CTD domains of BHG-CoV and HKU27, HKU28, and HKU29 are highly similar, but still a few amino acids have changed, and whether this change will affect their selection and utilization of receptors needs further research.

The estimation of differentiation date showed that tMRCA of HKU27, HKU28, and HKU29 was earlier than BHG-DCoV, which supports the possibility of cross-species transmission of them together with the host-switching event. In addition, the host–virus mapping model constructed in this study also supported that the origin of mammalian DCoVs was bird DCoVs (Figure 8). However, when the host transfer event appears at the node of the virus tree, its descendant node is transferred to a branch of the host tree which is not ancestrally related (neither an ancestor nor a descendant of the host) to the host (Santichaivekin et al., 2021). For the host transfer event, we could only explain the possible source through the tip (or the node near tip) of the virus tree. Therefore, whether the DCoV in houbara directly spreading to white-rumped snowfinch (*Montifringilla taczanowskii*), sparrow (*Passer montanus*), and munia (*Lonchura striata*), or their common ancestor, was not clear. Of note, the evolutionary relationship between the ancestors of *Night heron coronavirus HKU19*, *Wigeon coronavirus HKU20*, and their hosts was not clear due to the lower support value, and the host transfer from houbara to thrush (*Turdus hortulorum*) also lacked sufficient support.

It was believed previously that the host of DCoVs was limited to avian and swine only, but three child cases of acute undifferentiated febrile illness positive for porcine DCoV were reported in Haiti recently (Lednicky et al., 2021), raising the concern about transmission and pathogenicity of DCoV to humans, especially during long-term and close contact. Since *White-eye coronavirus HKU16* like coronavirus has been found in six orders of birds, suggesting the active interspecies transmission ability of *White-eye coronavirus HKU16* like coronavirus, ornithological studies have shown that the black-headed gulls in Yunan usually migrate for long distance from Russia, Mongolia, and Xinjiang Uygur Autonomous Region (Gan et al., 2019). In the large areas for migration, black-headed gulls contact a lot of people and animals, and the BHG-DCoVs carried by them may transmit to other birds including poultry fed in human communities, causing poultry epidemics and even human infections. Therefore, it is necessary to continue the monitoring of this DCoV.

Alphacoronavirus and betacoronavirus mainly infect mammals and have been reported to cause human diseases and even worldwide pandemics. Thus, the existence of alphacoronavirus and betacoronavirus in wild and domestic animals has been widely studied. In comparison, less attention has been paid to DCoV which mainly infects avian. However, increasing evidence indicates that DCoV is capable of causing diseases in poultry, swine, and even humans. Considering the widespread birds and rapid mutation of DCoV, interspecies transmission of DCoV may probably cause epidemics of humans and domestic animals. Thus, people should not omit the potential threat of DCoV to public health and animal husbandry industry. Our identification of DCoVs in black-headed gulls, the common birds with frequently long-distance and transboundary migration, further substantiates the importance of monitoring DCoVs in the nature.

## Data Availability Statement

The datasets presented in this study can be found in online repositories. The names of the repository/repositories and accession number(s) can be found below: https://www.ncbi.nlm.nih.gov/genbank/, OL311150, OL311151, and OL311152.

## Author Contributions

K-KC, Z-JZ, Y-ZZ, and X-YG contributed to the conception and design of the study. K-KC and LG collected the samples. Z-JZ organized the database. K-KC and Z-JZ performed the data analysis. K-KC and YQ wrote the first draft of the manuscript. All authors contributed to manuscript revision, read, and approved the submitted version.

## Conflict of Interest

The authors declare that the research was conducted in the absence of any commercial or financial relationships that could be construed as a potential conflict of interest.

## Publisher’s Note

All claims expressed in this article are solely those of the authors and do not necessarily represent those of their affiliated organizations, or those of the publisher, the editors and the reviewers. Any product that may be evaluated in this article, or claim that may be made by its manufacturer, is not guaranteed or endorsed by the publisher.

## Footnotes

1
http://timetree.org/
