# Supplementary material for: Characterization of Deltacoronavirus in Black-Headed Gulls (Chroicocephalus ridibundus) in South China Indicating Frequent Interspecies Transmission of the Virus in Birds
Source: Front Microbiol. 2022 May 12;13:895741. doi: 10.3389/fmicb.2022.895741 (PMC9133700; doi:10.3389/fmicb.2022.895741)
Supplement: Supplementary file 1 [file Data_Sheet_1.PDF]

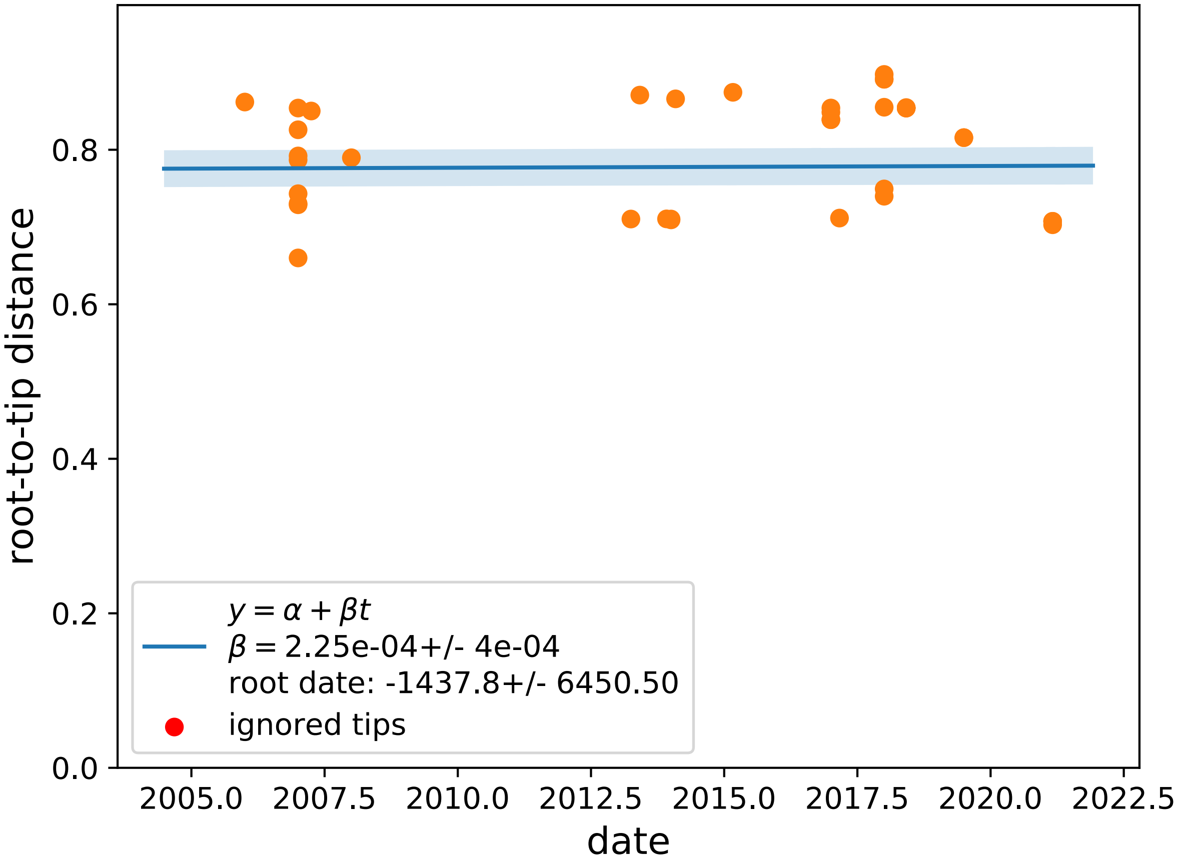

Figure S1. The regression analysis of samping date and root-to-tip distances. The horizontal axis represents the sampling time of the strains, and the vertical axis represents the genetic distance from the tip of each branch to the root. β value represents the evolution rate.
