# Supplementary material for: Characterization of Deltacoronavirus in Black-Headed Gulls (Chroicocephalus ridibundus) in South China Indicating Frequent Interspecies Transmission of the Virus in Birds
Source: Front Microbiol. 2022 May 12;13:895741. doi: 10.3389/fmicb.2022.895741 (PMC9133700; doi:10.3389/fmicb.2022.895741)
Supplement: Supplementary file 2 [file Data_Sheet_2.PDF]

```
                          *        20         *        40         *        60         *        80         *
Hu-PDCoV     : MQRALLI-MTLLCLVRAKFADDLLDLLTFPGAHRFLHKLTSNSSSFYSR-A---------NNFDVGVLP-GYPTENVNL--FSPLTNSTLPING :  80
PDCoV-HKU15  : MQRALLI-MTLLCLVRAKFADDLFDLLTFPGAHRFLHKPTRNSSSLYSRAN---------NNFDVGVLP-GYPTKNVNL--FSPLTNSTLPING :  81
A1CCoV       : MMQ------------------------------------------------------------------------------------------- :   3
HKU17-USA    : MQRTVLI-LLLLGTTHAKLADDILDVLTFPGAHRFLHKYSK--SVLQPRDS---------DNFDVGKMP-GFATKRFDL--FSPLTNTTLPING :  79
HNU4-1       : MQSIVLV-LMLSVVTASPLADKILDFLTFPGAHDYLR------QSPDPKIG---------EERCFRVLPGGYIPANFTFPQWFVLTNSSTFLNG :  78
HNU4-2       : MQSIVLV-LMLSVVTASPLADKILDFLTFPGAHDYLR------QSPDPKIG---------EERCFRVLPGGYIPANFTFPQWFVLTNSSTFLNG :  78
HNU4-3       : MQSIVLV-LMLSVVTASPLADKILDFLTFPGAHEYLR------QSPDPKIG---------EERCFRVLPGGYIPANFTFPQWFVLTNSSTFLNG :  78
HKU27        : MQSIILV-TILSAVTASPIADKILDFLTFPGAHQHLR------QSPEPKGG---------RERCFKVLQGGYIPDNFTFPNWFVLTNGSTFLNG :  78
HKU28        : MQSIILV-TILSAATASPLADKILDFLTFPGAHQYLK------QTPQPKLG---------RERCFNVLPGGYIPDSFGFPSWFVLTNSSTFLNG :  78
HKU29        : MQSIILV-TILSAATASPLADKILDFLTFPGAHQYLK------QTPQPKLG---------RERCFNVLPGGYIPDSFGFPSWFVLTNSSTFLNG :  78
HKU16        : MQRIILISTILYCARALTLADKMLDLLTFPGAHHYFR---GDLQTLHSRISAESYSVNPYDQYNYQTDSDYYINKSVHL--IAPLTNLTLPISG :  89

                  100         *       120         *       140         *       160         *       180
Hu-PDCoV     : LHRSYQPLMLNC---LTKITNHTLSMY------LLPSEIQTYSCGGA--MVKYQT----HDAVRIILDLTVTDHIS----VEVVGQRGENYVFV : 155
PDCoV-HKU15  : LHRSYQPLMLNC---LTKITNHTLSMY------LLPSEIQTYSCGGA--MVKYQT----HDAVRIILDLTATDHIS----VEVVGQHGENYVFV : 156
A1CCoV       : --------------------------------------------------------------VRIILDLTATDHIS----VEVVGQDGENYVFV :  31
HKU17-USA    : LHRSYQPLMLSC---FTDITNETLSMH------LQPSNEATYNCGGE--LVKYQT----HDALRLILNLT----EASSAYIKVFGPDNLFYVFV : 154
HNU4-1       : EYTMEQPLFANAHL-CTWQSNQQYRRYGFNNSCLLPADSCNPKHNGNPLFKGWNNTVGFSDTIRINVNITSYDYDSYAGFINISLIDGSYISIQ : 171
HNU4-2       : EYTMEQPLFANAHL-CTWQSNQQYRRYGFNNSCLLPADSCNPKHNGNPLFKGWNNTVGFSDTIRINVNITSYDYDSYAGFINISLIDGSYISIQ : 171
HNU4-3       : EYTMEQPLFANAHL-CTWQSNQQYRRYGFNNSCLLPADSCNPKHNGNPLFKGWNNTVGFSDTIRINVNITSYDYHSYAGFINISLIDGSYISIQ : 171
HKU27        : EYTMQQPLFANAHI-CTWQSNPRYRRYGFNNSCLLPADGCTPNFNGSPLFKGWNNTIGFSDTIRINVNISSYDYSSYAGFINISQGDGSYISIQ : 171
HKU28        : EYTMEQPLFANAHL-CTWQSNQQYRRYGFNNSCLLPADSCNPKHNGNSLFKGWNNTVGFSDTIRINVNITSYDYDSYAGFINISQSDGSYISIQ : 171
HKU29        : EYTMEQPLFANAHL-CTWQSNQQYRRYGFNNSCLLPADSCNPKHNGSSLFKGWNNTVGFSDTIRINVNITSYDYDSYAGFINISQSDGSYISIQ : 171
HKU16        : LHRSMQPLRVGCIFGASNKIDQGFTISGM----TYPLAYCVPPF--------YQVTNVTYDAMRLLFAFA--DLNSTGDFLRINTKTMGMLNVS : 169

               *       200         *       220         *       240         *       260         *       280
Hu-PDCoV     : CSEQFN---YTTALHNSTFFSLNSELYCFTNNT--------YLGILPPDLTDFTVYRTGQFYANGYLLGTLPITVNYVRLYRGHLSANSAHFAL : 238
PDCoV-HKU15  : CSEQFN---YTTALHNSTFFSLNSELYCFTNNT--------YLGILPPDLTDFTVYRTGQFYANGYLLGTLPITVNYVRLYRGHLSANNAHFAL : 239
A1CCoV       : CSEQFN---YTTALHNSTFFSLNSELYCFTNNT--------YLGILPPDLTDFTVYRTGQFYANGYLLGTLPITVNYVRLYRGHLAANSAHFAL : 114
HKU17-USA    : CSENFN---YTTALQNSTFFSANKQLYCFTNDT--------YLGILPPDLTDITVYRTGQFYANGYLFGTLPITVSKVRLYKGFLPSHSAHFAL : 237
HNU4-1       : CTNDSDPEKLSVARSVLPFTYIGKTIYCFANFTQQKTSYIQFMGVLPPHVAELAFDRTGNIYINGYRYFQIS-TIDYIQ-FGLRFNTSNIHFSV : 263
HNU4-2       : CTNDSDPEKLSVARSVLPFTYIGKTIYCFANFTQQKTSYIQFMGVLPPHVAELAFDRTGNIYINGYRYFQIS-TIDYIQ-FGLRFNTSNIHFSV : 263
HNU4-3       : CTNDSDPEKLSVARSVLPFTYIGKTIYCFANFTQQKTSYIQFMGVLPPDVAELAFDRTGNIYINGYRYFQIS-TIDYIQ-FGLRFNTSNIHFSV : 263
HKU27        : CTNDSDPSTLSLARSVLPLTYIGKTIYCFANITQQQTSYIEFMGVLPPYVAELSFDRTGNIYINGYRYFQIS-TINYIE-FGLAFNSSNIHFSV : 263
HKU28        : CTNDSDPSTLSVARSVLPLTYIGKTIYCFANFTQQQTSYIQFMGVLPPHVAELAFDRTGNIYINGYRYFQIS-TIDYIQ-FGLAFNNSPIHFSV : 263
HKU29        : CTNDSDPSTLSVARSVLPLTYIGKTIYCFANFTQQQTSYIQFMGVLPPHVAELAFDRTGNIYINGYRYFQIS-TIDYIQ-FGLAFNNSPIHFSV : 263
HKU16        : CSASPTP--LGHQDADRTFYGYNKQLYCYLDTP----AGMQYMGPLPANLTEITLFRTGQIYTNGFHLGTIPSELTYVYLDKLAFQNKTVCM-M : 256

                         *       300         *       320         *       340         *       360         *
Hu-PDCoV     : ANLTDTLITLTNT--TISQITYCDKSVVDSIACQRSSHEVEDGFYSDPKSAVRARQRTIVTLPKLPELEVVQLNISAHMD--FGEARLD-SVTI : 327
PDCoV-HKU15  : ANLTDTLITLTNT--TISQITYCDKSVVDSIACQRSSHEVEDGFYSDPKSAVRARQRTIVTLPKLPELEVVQLNISAHMD--FGEARLD-SVTI : 328
A1CCoV       : ANLTDTLITLTNT--TISQITYCDKSVVDSIACQRSSHQVEDGFYSDPKSAVRARQRTIVTLPKLPELEVVQLNISAHMD--FGEARLD-SVTI : 203
HKU17-USA    : ANLTDTLITLVNT--TITNITYCDSSVISSIACQRSSHAVEDGFYSDPATAANALQRTVVTLPKLPEMEVVQINITAHME--FGQTKLDH-ISI : 326
HNU4-1       : TWLNYTRVHLNTQEGVIKKIMYCDSD-YNSLTCDMNTFSLPDGVYSFSSLQRDLVPQTFVTLPVYANHSYLVLNTSYTVGSCVNCPPIQHSIHI : 356
HNU4-2       : TWLNYTRVHLNTQEGVIKKIMYCDSD-YNSLTCDMNTFSLPDGVYSFSSLQRDLVPQTFVTLPVYANHSYLVLNTSYTVGSCVNCPPIQHSIHI : 356
HNU4-3       : TWLNYTRVHLNTQEGVIKKIMYCDSD-YNSLTCDMNTFSLPDGVYSFSSLQRDLVPQTFVTLPVYANHSYLVLNTSYTVGSCVNCPPIQHSIHI : 356
HKU27        : TWLNYTRVHLNTQQGVIKNIMYCDSD-YNALTCDMNTFSLPDGVYSFSSLQRDLVPQTFVTLPVYANHSYLVLNTSYTVGLCVNCPPIQHSIYI : 356
HKU28        : TWLNYTRVHFNTQEGVIKKIMYCDSD-YNSLTCDMNTFSLPDGVYSFSSLQMDLVPQTFVTLPVYANHSDLVLNTSYTVGSCVNCPPIQHSIYI : 356
HKU29        : TWLNYTRVHFNTQEGVIKKIMYCDSD-YNSLTCDMNTFSLPDGVYSFSSLQMDLVPQTFVTLPVYANHSDLVLNTSYTVGSCVNCPPIQHSIYI : 356
HKU16        : ANLTDTLITLNHT--VIQQVTYCEKDAVQALACQQSTHQLQDGFYSDPAPAVNNLPKTLVTLPKIAESSTLQINVSATYS--YGSASGSIKLSY : 346

                 380         *       400         *       420         *       440         *       460         *
Hu-PDCoV     : NG--NTSYCATKPYFRLETNFMC-TGCTMNLRT----DTCSFDLSAVNNGMSFSQFCLST-ESGACEMKIIVTY--VWNYLLRQ--RLYVTAVE : 409
PDCoV-HKU15  : NG--NTSYCVTKPYFRLETNFMC-TGCTMNLRT----DTCSFDLSAVNNGMSFSQFCLST-ESGACEMKIIVTY--VWNYLLRQ--RLYVTAVE : 410
A1CCoV       : NG--NTSYCVTKPYFRLETNFLC-RGCTMSLRT----DTCSFDLSAVNNGMSFSQFCLST-ESGACEMKIIVTY--VWNYLLRQ--RLYVTAVE : 285
HKU17-USA    : NG--NTTYCVAKPYFKLEMNFYC-ASCTISLRT----DTCNFDLFAVNNGMSFSQFCIST-ESGACEIKLIATY--VWNHLMRQ--RLYVTAVE : 408
HNU4-1       : EGAVNDTYCVNSKQFTLKINSHVDATYSQYFKTVFTAGTCPFTFPNVNNFLSFESFCISTVNTGGCTIHVQK----VWNSVVHTFATLYVSYQE : 446
HNU4-2       : EGAVNDTYCVNSKQFTLKINSHVDATYSQYFKTVFTAGTCPFTFPNVNNFLSFESFCISTVNTGGCTIHVQK----VWNSVVHTFATLYVSYQE : 446
HNU4-3       : EGAVNDTYCVNSKQFTLKINSHVDATYSQYFKTVFTAGTCPFTFPNVNNFLSFESFCISTVNIGGCTIHVQK----VWNSVVHTFATLYVSYQE : 446
HKU27        : EGAVNDTYCVNSKQFTLKINSHVEATYSQYFKTVFTAGTCPFTFPNVNNFLSFESFCISTVNNGGCTIHVQK----VWNSVVHTFATLYVSYQE : 446
HKU28        : EGAVNDTYCVNSKQFTLKINSHVDAKYSQYFKTVFTAGTCPFTFPNVNNFLSFESFCISTVNNGGCTIHVQK----VWNSVVHTFATLYVSYQE : 446
HKU29        : EGAVNDTYCVNSKQFTLKINSHVDAKYSQYFKTVFTAGTCPFTFPNVNNFLSFESFCISTVNNGGCTIHVQK----VWNSVVHTFATLYVSYQE : 446
HKU16        : NGSSNNSHCVQTPYFKLEQNLVCSGGCSVRIETL----TCPFDLNAVSNGMSFQQFCVST-VSGQCSMQAIVNTGQPWGYV-TS--TLYVTYVE : 432

                         480         *       500         *       520         *       540         *       560
Hu-PDCoV     : GQTHTGT--TSVHATDTSSVITDVCTDYTIYGVSGTGIIKPSDLLLHNGIAFTSPTGELYAFKNITTGKTLQVLPCETPSQLIVINNTVVGAIT : 501
PDCoV-HKU15  : GQTHTGT--TSVHATDTSSVITDVCTDYTIYGVSGTGIIKPSDLLLHNGIAFTSPTGELYAFKNITTGKTLQVLPCETPSQLIVINNTVVGAIT : 502
A1CCoV       : GQTHTGT--TAVHATDTSSVVTDVCTDYTIYGVSGTGIIKPSDLLLHNGIAFTSPTGELYAFKNITTGKTLQVLPCETPSQLIVINNTVVGAIT : 377
HKU17-USA    : GQTHTGV--NSVYAIDTSSVVTGVCTDYTIYGISGKGVIQPSELSLHNGIAFTSPTGELYAFKNITTGKAFQVLPCDTPSQLIVINNTIVGAIT : 500
HNU4-1       : GNSIHAMPQPATGTSDISTVHLDVCSRYNIYGKTGNGIIKLTNQSYIAGLYYTSPSGDLLAFKNVTTQQVYSVTPCRPSSQVAVYNGSIIAAFT : 540
HNU4-2       : GNSIHAMPQPATGTSDISTVHLDVCSRYNIYGKTGNGIIKLTNQSYIAGLYYTSPSGDLLAFKNVTTQQVYSVTPCRPSSQVAVYNGSIIAAFT : 540
HNU4-3       : GNSIHAMPQPATGTSDISTVHLDVCSRYNIYGKTGNGIIKLTNQSYIAGLYYTSPSGDLLAFKNVTTQQVYSVTPCRPSSQVAVYNGSIIAAFT : 540
HKU27        : GNSIHAMPQPATGTSDISTVHLDVCSRYNIYGKTGNGIIRLTNQSYIAGLYYTSPSGDLLAFKNVTTQQVYSVTPCRPSSQVAVYNGSIIAAFT : 540
HKU28        : GNSIHAMPKPATGTSDISTVYLDVCSRYNIYGKTGNGIIRLTNQSYIAGLYYTSPSGDLLAFKNVTTQQVYSVTPCRPSSQVAVYNGSIIAAFT : 540
HKU29        : GNSIHAMPKPATGISDISTVYLDVCSRYNIYGKTGNGIIRLTNQSYIAGLYYTSPSGDLLAFKNVTTQQVYSVTPCRPSSQVAVYNGSIIAAFT : 540
HKU16        : GQSFTGT--SSDQIEDLTVLHLDQCTSYTIYGVSGTGVITLSDLQLPHGITFRAANGELSAFKNTTTGDVYTIQPCSLPAQLAIIDSTIVGAIT : 524

                   *       580         *       600         *       620         *       640         *       6
Hu-PDCoV     : SSNSTENNRFTTTIVTPTFFYSTNATTFNCTKPVLSYGPISVCSDGAIAGTSTLQNTRPSIVSLYDGEVEIPSAFSLSVQTEYLQVQAEQVIVD : 595
PDCoV-HKU15  : SSNSTENNRFTTTIVTPTFFYSTNATTFNCTKPVLSYGPISVCSDGAIAGTSTLQNTRPSIVSLYDGEVEIPSAFSLSVQTEYLQVQAEQVIVD : 596
A1CCoV       : SSNSTENNRFTTTIVTPTFFYSTNATTFNCTKPVLSYGPISVCSDGAIAGTSTLQNTRPSIVSLYDGEVEIPSAFSLSVQTEYLQVQAEQVIVD : 471
HKU17-USA    : STNK-TVSGFTKIIPTPTFYYSTNATIFNCTNPALSYGPISVCADGSIVETNTLQNSRPSIVSLYDGEVEIPSDFTLSVQTEYLQVQSEQVIVD : 593
HNU4-1       : STENVTIETLTYSRATPMFYYHSIGNE-SCNTPVVSFGSIGVCSGGGLHFVDSASNEFTNVVPISTQNISIPVNFTVSIQSEYIQIEQQPVTVD : 633
HNU4-2       : STENVTIETLTYSRATPMFYYHSIGNE-SCNTPVVSFGSIGVCSGGGLHFVDSASNEFTNVVPISTQNISIPVNFTVSIQSEYIQIEQQPVTVD : 633
HNU4-3       : STENVTIETLTYSRATPMFYYHSIGNE-SCNTPVVSFGSIGVCSGGGLHFVDSASNEFTNVVPISTQNISIPVNFTVSIQSEYIQIEQQPVTVD : 633
HKU27        : STENVAIPTLTYSRATPMFYYHSIGND-SCDTPVISFGSIGVCSGGGLHFVDSASNEFTNVVPISTQNISIPVNFTVSIQSEYIQIEQQPVTVD : 633
HKU28        : STENVTIETLTYSRATPMFYYHSVGND-SCDAPVVSFGSIGVCSGGGLHFVDSASNEFTNVVPISTQNISIPVNFTVSIQSEYIQIEQQPVTVD : 633
HKU29        : STENVTIETLTYSRATPMFYYHSVGND-SCDAPVVSFGSIGVCSGGGLHFVDSASNEFTNVVPISTQNISIPVNFTVSIQSEYIQIEQQPVTVD : 633
HKU16        : STN--ESYGFSNTIVTPTFYYSTNATS-NCTAPKISYGELGVCADGSIGAVSQLQDSKPSIVPLYTGEIEIPASFKLSVQTEYLQVQTEQVVID : 615
```

```
                60        *       680         *       700         *       720         *       740         *
Hu-PDCoV     : CPQYVCNGNSRCLQLLAQYTSACSNIEAALHSSAQLDSREIINMFQTSTQSLQLANITNFK-GD---YNFSSILTTRLG-GRSAIEDLLFNKVV :  684
PDCoV-HKU15  : CPQYVCNGNSRCLQLLAQYTSACSNIEAALHSSAQLDSREIINMFQTSTQSLQLANITNFK-GD---YNFSSILTTRLG-GRSAIEDLLFNKVV :  685
AlCCoV       : CPQYVCNGNSRCLQLLAQYTSACSNIEAALHSSAQLDSREIINMFQTSTQSLQLANITNFK-GD---YNFSSILTTRVG-GRSAIEDLLFNKVV :  560
HKU17-USA    : CPQYVCNGNSRCLRLLAQYTSACSNIESALHSSAQLDSREITTMFQTSSQSLELANITNFK-GD---YNFSAILTNNLG-GKSAIEDLLFNKVV :  682
HNU4-1       : CRQYVCNGNERCLQLLMQYTSACSSIEQALSLNARLEAADIQTMLTYSPDTLELANITYFQSGDGLDYNLTNILPKKYQ-GRSPIEDILFDKVV :  726
HNU4-2       : CRQYVCNGNERCLQLLMQYTSACSSIEQALSLNARLEAADIQTMLTYSPDTLELANITYFQSGDGLDYNLTNILPKKYQ-GRSPIEDILFDKVV :  726
HNU4-3       : CRQYVCNGNERCLQLLMQYTSACSSIEQALSLNARLEAADIQTMLTYSPDTLELANITYFQSGDGLDYNLTNILPKKYQ-GRSPIEDILFDKVV :  726
HKU27        : CRQYVCNGNERCLQLLMQYTSACSSIEQALSLNARLEAADIQTMLTYSPDTLKLANITNFESDD-VNYNLTNILPKKYQ-GRSPIEDILFDKVV :  725
HKU28        : CRQYVCNGNERCLQLLMQYTSACSSIEQALSLNARLEAADIQTMLTYSPETLKLANITNFESGD-VNYNLTNILPKKYQ-GRSPIEDILFDKVV :  725
HKU29        : CRQYVCNGNERCLQLLMQYTSACSSIEQALSLNARLEAADIQTMLTYSPETLKLANITNFESGD-VNYNLTNILPKKYQ-GRSPIEDILFDKVV :  725
HKU16        : CPKYVCNGNPRCLQLLAQYTSACSNIESALHSSAQLDSREITMMFQTSSQSVELANITNFQ-GD---YNFSMILPTLPGKDRSAIEDLLFDKVV :  705

                   760         *       780         *       800         *       820         *       840
Hu-PDCoV     : TSGLGTVDQDYKACSRDMAIADLVCSQYYNGIMVLPGVVDAEKMAMYTGSLTGAMVFGGLTAAAAIPFATAVQARLNYVALQTNVLQENQKILA :  778
PDCoV-HKU15  : TSGLGTVDQDYKACSRDMAIADLVCSQYYNGIMVLPGVVDAEKMAMYTGSLTGAMVFGGLTAAAAIPFATAVQARLNYVALQTNVLQENQKILA :  779
AlCCoV       : TSGLGTVDQDYKSCSRDMAIADLVCSQYYNGIMVLPGVVDAEKMAMYTGSLTGAMVFGGLTAAAAIPFATAVQARLNYVALQTNVLQENQKILA :  654
HKU17-USA    : TNGLGTVDQDYKSCSKDMAIADLVCSQYYNGIMVLPGVVDAQKMAMYTGSLTGAMVFGGLTAAAAIPFATAVQARLNYVALQTNVLQENQKILA :  776
HNU4-1       : TNGLGTVDQDYKACTNGVFLADLACAQYYNGIMVLPGVADPATMAMYTASLTGGMVFGGLTSAAAIPFSLAVQSRLNYVALQTDVLQQNQKILA :  820
HNU4-2       : TNGLGTVDQDYKACTNGVFLADLACAQYYNGIMVLPGVADPATMAMYTASLTGGMVFGGLTSAAAIPFSLAVQSRLNYVALQTDVLQQNQKILA :  820
HNU4-3       : TNGLGTVDQDYKACTNGVFLADLACAQYYNGIMVLPGVADPATMAMYTASLTGGMVFGGLTSAAAIPFSLAVQSRLNYVALQTDVLQQNQKILA :  820
HKU27        : TNGLGTVDQDYKACTNGVFLADLACAQYYNGIMVLPGVADPAKMAMYTASLTGGMVFGGFTAAAAIPFSLAVQSRLNYVALQTDVLQQNQKILA :  819
HKU28        : TNGLGTVDQDYKACTNGVFLADLACAQYYNGIMVLPGVADPAKMAMYTASLTGGMVFGGLTSAAAIPFSLAVQSRLNYVALQTDVLQQNQKILA :  819
HKU29        : TNGLGTVDQDYKACTNGVFLADLACAQYYNGIMVLPGVADPAKMAMYTASLTGGMVFGGLTSAAAIPFSLAVQSRLNYVALQTDVLQQNQKILA :  819
HKU16        : TNGLGTVDQDYKSCSKGIAVADLVCAQYYNGIMVLPGVVDAEKMAMYTGSLTGAMVFGGLTAAAAIPFSTAVQARLNYVALQTNVLQENQKILA :  799

                 *       860         *       880         *       900         *       920         *       940
Hu-PDCoV     : ESFNQAVGNISLALSSVNDAIQQTSEALNTVAIAIKKIQTVVNQQGEALSHLTAQLSNNFQAISTSIQDIYNRLEEVEANQQVDRLITGRLAAL :  872
PDCoV-HKU15  : ESFNQAVGNISLALSSVNDAIQQTSEALNTVAIAIKKIQTVVNQQGEALSHLTAQLSNNFQAISTSIQDIYNRLEEVEANQQVDRLITGRLAAL :  873
AlCCoV       : ESFNQAVGNISLALSSVNDAIQQTSEALNTVAIAIKKIQTVVNQQGEALSHLTAQLSNNFQAISTSIQDIYNRLEEVEANQQVDRLITGRLAAL :  748
HKU17-USA    : ESFNQAVGNISLALSSVNDAIQQTSEALNTVANAINKIQTVVNQQGEALSHLTAQLSNNFQAISTSIQDIYNRLEEVEANQQVDRLITGRLAAL :  870
HNU4-1       : DSFNSAMGNISLAFKEVSEGLSQVSGAITTVANALTKIQTVVNSQGQALATLTEQLANNFQAISASIADIYNRLNQLEADAQVDRLITGRLAAL :  914
HNU4-2       : DSFNSAMGNISLAFKEVSEGLSQVSGAITTVANALTKIQTVVNSQGQALATLTEQLANNFQAISASIADIYNRLNQLEADAQVDRLITGRLAAL :  914
HNU4-3       : DSFNSAMGNISLAFKEVSEGLSQVSGAITTVANALTKIQTVVNSQGQALATLTEQLANNFQAISASIADIYNRLNQLEADAQVDRLITGRLAAL :  914
HKU27        : DSFNSAMGNISLAFKEVSEGLSQVSGSITTVANALIKIQTVVNSQGQALATLTEQLANNFQAISASIADIYNRLNQLEADAQVDRLITGRLAAL :  913
HKU28        : DSFNSAMGNISLAFKEVSEGLSQVSGAITTVANALTKIQTVVNSQGQALATLTEQLANNFQAISASIADIYNRLNQLEADAQVDRLITGRLAAL :  913
HKU29        : DSFNSAMGNISLAFKEVSEGLSQVSGAITTVANALTKIQTVVNSQGQALATLTEQLANNFQAISASIADIYNRLNQLEADAQVDRLITGRLAAL :  913
HKU16        : ESFNQAVGNISLALSNVNTAIQQTSEALLTVSNAINKIQTVVNQQGEALAHLTAQLSQNFQAISTSIQDIYNRLDQIQADQQVDRLITGRLAAL :  893

                         *       960         *       980         *       1000        *       1020        *
Hu-PDCoV     : NAYVTQLLNQMSQIRQSRLLAQQKINECVKSQSSRYGFCGNGTHIFSLTQTAPNGIFFMHAVLVPNKFTRVNASAGICVDNIKGYSLQPQLILY :  966
PDCoV-HKU15  : NAYVTQLLNQMSQIRQSRLLAQQKINECVKSQSPRYGFCGNGTHIFSLTQTAPNGIFFMHAVLVPNKFTRVNASAGICVDNTRGYSLQPQLILY :  967
AlCCoV       : NAYVTQLLNQMSQIRQSRLLAQQKXNECVKSQSSRYGFCGNGTHIFSLTQAAPNGIFFMHAVLVPNKFTRVNASAGICVDNTRGYSLQPQLILY :  842
HKU17-USA    : NAYVTQLLNQMSQIRQSRLLAQQKINECVKSQSSRYGFCGNGTHLFSITQAAPNGIFFMHAVLVPTKFARVNASAGICVDNTKGYSLQPQLILY :  964
HNU4-1       : NAFVTQTLSKLAEVRQARQLANDKINECVKSQSSRYGFCGNGTHLFSMVNAAPYGFVFFHTVLLPTQYTTVQAYSGICQNGKAMALKDPSLALF : 1008
HNU4-2       : NAFVTQTLSKLAEVRQARQLANDKINECVKSQSSRYGFCGNGTHLFSMVNAAPYGFVFFHTVLLPTQYTTVQAYSGICQNGKAMALKDPSLALF : 1008
HNU4-3       : NAFVTQTLSKLAEVRQARQLANDKINECVKSQSSRYGFCGNGTHLFSMVNAAPYGFVFFHTVLLPTQYTTVQAYSGICQNGKAMALKDPSLALF : 1008
HKU27        : NAFVTQTLSKLAEVRQARQLANDKINECVKSQSSRYGFCGNGTHLFSMVNAAPYGFVFFHTVLPPTQYTTVQAYSGICQNGKAMALKDPSLALF : 1007
HKU28        : NAFVTQTLSKLAEVRQARQLANDKINECVKSQSSRYGFCGNGTHLFSMVNAAPYGFVFFHTVLLPTQYTTVQAYSGICQNGKAMALKDPSLALF : 1007
HKU29        : NAFVTQTLSKLAEVRQARQLANDKINECVKSQSSRYGFCGNGTHLFSMVNAAPYGFVFFHTVLLPTQYTTVQAYSGICQNGKAMALKDPSLALF : 1007
HKU16        : NAYVTQLLNKLSQVRQSRILAEQKINECVKSQSSRYGFCGNGTHLFSLTQAAPNGIFFMHAVLVPQTFQPVVAYAGICVDGY-GYSLQPQLVLY :  986

                 1040        *       1060        *       1080        *       1100        *       1120
Hu-PDCoV     : QFNNSWRVTPRNMYEPRLPRQADFIQLTDCSVTFYNTTAANLPNIIPDIIDVNQTVSDIIDNLPTATPPQWDVGIYNNTILNLTVEINDLQERS : 1060
PDCoV-HKU15  : QFNNSWRVTPRNMYEPRLPRQADFIQLTDCSVTFYNTTAANLPNIIPDVIDVNQTVSDIIDNLPTATPPQWDVGIYNNTILNLTVEINDLQERS : 1061
AlCCoV       : QFNNSWRVTPRNMYEPRLPRQADFIQLTDCSVTFYNTTAANLPNIIPDVIDVNQTVSDIIDNLPTATPPQWDVGIYNNTILNLTVEINDLQERS :  936
HKU17-USA    : QYNDSWRVTPRNMYEPRIPRAADFIPLVDCSVTFYNTTASDLPNIIPDIVDVNQTVSEILGNITSPQLPSLSVDYYNNTILNLTLEIDDLQERS : 1058
HNU4-1       : RGTNHYLVTPRNMYQPRQASMSDFVTIESCTVTYLNLTDTTIDAVIPDYVDVNKTVEDILNNLPNYTKPDLDLGRYNNTILNLTTEINDLNGRA : 1102
HNU4-2       : RGTNHYLVTPRNMYQPRQASMSDFVTIESCTVTYLNLTDTTIDAVIPDYVDVNKTVEDILNNLPNYTKPDLDLGRYNNTILNLTTEINDLNGRA : 1102
HNU4-3       : RGTNHYLVTPRNMYQPRQASMSDFVTIESCTVTYLNLTDTTIDAVIPDYVDVNKTVEDILNNLPNYTKPDLDLGRYNNTILNLTTEINDLNGRA : 1102
HKU27        : RGTNHYLVTPRNMYQPRQASMSDFVTIESCTVTYLNLTDTTIDAVIPDYVDVNKTVEDILNNLPNYTKPDLDLGRYNNTILNLTNEIDDLNGRA : 1101
HKU28        : RGTSHYLVTPRNMYQPRQASISDFVTIESCTVTYLNLTDTTIDAVIPDYVDVNKTVEDILNNLPNYTKPDLDLGRYNNTILNLTTEINDLNGRA : 1101
HKU29        : RGTSHYLVTPRNMYQPRQASISDFVTIESCTVTYLNLTDTTIDAVIPDYVDVNKTVEDILNNLPNYTKPDLDLGRYNNTILNLTTEINDLNGRA : 1101
HKU16        : NLNDSYRITPRNMFEPRTPTQSVFIPLTTCSVDFVNVTANNVSIIIPDYVDVNKTVSDIINGLPNYSYPELSLDRFNHTILNLSQEIEDLQIRS : 1080

                 *       1140        *       1160        *       1180        *       1200        *       1220
Hu-PDCoV     : KNLSQIADRLQNYIDNLNNTLVDLEWLNRVEIYLKWPWYIWLAIALALIAFVTILITIFLCTGCCGGCFGCCGGCFGLFSKKKRYTDDQPTPS- : 1153
PDCoV-HKU15  : KNLSQIADRLQNYIDNLNNTLVDLEWLNRVETYLKWPWYIWLAIALALIAFVTILITIFLCTGCCGGCFGCCGGCFGLFSKKKRYTDDQPTPS- : 1154
AlCCoV       : KNLSQIADRLQNYIDNLNNTLVDLEWLNRVETYLKWXWYIWLAIALALIAFVTILITIFLCTGCCGGCFGCCGGCFGLFSKKKRYTDDQPTPS- : 1029
HKU17-USA    : KNLSLIADRLQIYIDNLNNTLVDLEWLNRVETYLKWPWYVWLAIFLAIAAFVAILITIFLCTGCCGGCFGCCGGCFGLFSKKKRITDDQPTPS- : 1151
HNU4-1       : ENLTQIVENLQEYIDKINATIVDLEWLNRVETYIKWPWWVWLLIVLAIAAFACIVVTIFLCTGCCGGCFGCCGACFGLFSKK-RHLNTEPTPVS : 1195
HNU4-2       : ENLTQIVENLQEYIDKINATIVDLEWLNRVETYIKWPWWVWLLIVLAIAAFACIVVTIFLCTGCCGGCFGCCGACFGLFSKK-RHLNTEPTPVS : 1195
HNU4-3       : ENLTQIVENLQEYIDKINATIVDLEWLNRVETYIKWPWWVWLLIVLAIAAFACIVVTIFLCTGCCGGCFGCCGGCFGLFSKK-RHLNTEPTPVS : 1195
HKU27        : ENLTQIVANLQDYIDKINATIVDLEWLNRVETYIKWPWWVWLLIVLAIAAFACIVVTIFLCTGCCGGCFGCCGGCFGLFSKK-RHLNTEPTPVS : 1194
HKU28        : ENLTQIVENLQEYIDKINATIIDLEWLNRVETYIKWPWWVWLLIVLAIAAFACIVVTIFLCTGCCGGCFGCCGGCFGLFSKK-RHLNTEPTPVS : 1194
HKU29        : ENLTQIVENLQEYIDKINATIIDLEWLNRVETYIKWPWWVWLLIVLAIAAFACIVVTIFLCTGCCGGCFGCCGGCFGLFSKK-RHLNTEPTPVS : 1194
HKU16        : QNLSATAELLQQYIDNLNNTLVDLEWLNRVETYLKWPWYIWLLIFLAIAAFATILVTIFLCTGCCGGCFGCCGGCFGLFSKK-RRLSSEPTPVS : 1173

Hu-PDCoV     : FKFKEW : 1159
PDCoV-HKU15  : FKFKEW : 1160
AlCCoV       : FKFKEW : 1035
HKU17-USA    : FKFKEW : 1157
HNU4-1       : FKLKEW : 1201
HNU4-2       : FKLKEW : 1201
HNU4-3       : FKLKEW : 1201
HKU27        : FKLKEW : 1200
HKU28        : FKLKEW : 1200
HKU29        : FKLKEW : 1200
HKU16        : FKLKEW : 1179
```

Figure S2. Multiple comparison of amino acid sequences of S protein of BHG-DCoV, FalCoV UAE-HKU27,HouCoV UAE-HKU28, PiCoV UAE-HKU29, HKU16, Hu-PDCoV(MW685622.1), PDCoV-HKU15(JQ065042.2), HKU17-USA(MG812377.1) and AlCCoV(EF584908.1).
