# Supplementary material for: Characterization of Deltacoronavirus in Black-Headed Gulls (Chroicocephalus ridibundus) in South China Indicating Frequent Interspecies Transmission of the Virus in Birds
Source: Front Microbiol. 2022 May 12;13:895741. doi: 10.3389/fmicb.2022.895741 (PMC9133700; doi:10.3389/fmicb.2022.895741)
Supplement: Supplementary file 3 [file Data_Sheet_3.PDF]

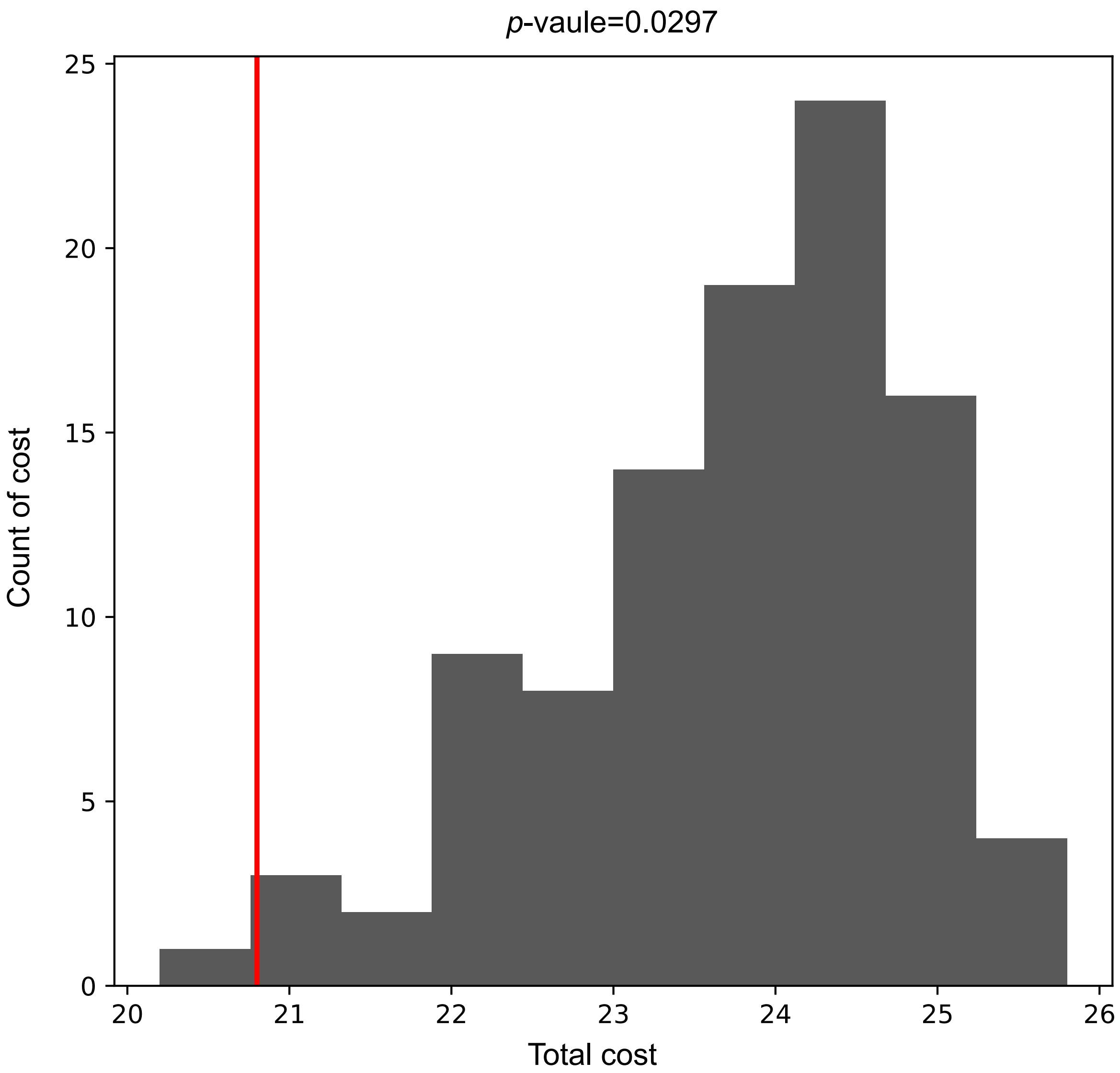

Figure S3 The distribution of costs of radom sample. The histogram represented the costs random sample, and the cost of original data was marked with a red line.
