# Supplementary material for: Characterization of Deltacoronavirus in Black-Headed Gulls (Chroicocephalus ridibundus) in South China Indicating Frequent Interspecies Transmission of the Virus in Birds
Source: Front Microbiol. 2022 May 12;13:895741. doi: 10.3389/fmicb.2022.895741 (PMC9133700; doi:10.3389/fmicb.2022.895741)
Supplement: Supplementary file 4 [file Data_Sheet_4.PDF]

Table S1. The identity (aa%) of S protein.

	Hu-PDCoV	PDCoV-HKU15	AlCCoV	HKU17-USA	HNU4-1	HNU4-2	HNU4-3	HKU27	HKU28	HKU29	HKU16
**Hu-PDCoV**		98.6207	87.4971	81.3734	45.0577	45.0577	45.2224	44.765	44.765	44.765	60.7679
**PDCoV-HKU15**	98.6207		87.7665	81.3734	45.14	45.14	45.3048	44.8475	44.765	44.765	60.9349
**AlCCoV**	87.4971	87.7665		74.5564	42.1433	42.1433	42.3089	41.9297	42.0125	42.013	57.6191
**HKU17-USA**	81.3734	81.3734	74.5564		45.462	45.462	45.6271	45.2519	45.3344	45.334	61.1715
**HNU4-1**	45.0577	45.14	42.1433	45.462		100	99.5837	93.9217	95.7535	95.587	46.4432
**HNU4-2**	45.0577	45.14	42.1433	45.462	100		99.5837	93.9217	95.7535	95.587	46.4432
**HNU4-3**	45.2224	45.3048	42.3089	45.6271	99.5837	99.5837		94.005	95.6703	95.504	46.5249
**HKU27**	44.765	44.8475	41.9297	45.2519	93.9217	93.9217	94.005		94.5	94.5	46.3993
**HKU28**	44.765	44.765	42.0125	45.3344	95.7535	95.7535	95.6703	94.5		99.833	46.8085
**HKU29**	44.765	44.765	42.0125	45.3344	95.587	95.587	95.5037	94.5	99.8333		46.8903
**HKU16**	60.7679	60.9349	57.6191	61.1715	46.4432	46.4432	46.5249	46.3993	46.8085	46.89	
