# Supplementary material for: Characterization of Deltacoronavirus in Black-Headed Gulls (Chroicocephalus ridibundus) in South China Indicating Frequent Interspecies Transmission of the Virus in Birds
Source: Front Microbiol. 2022 May 12;13:895741. doi: 10.3389/fmicb.2022.895741 (PMC9133700; doi:10.3389/fmicb.2022.895741)
Supplement: Supplementary file 5 [file Data_Sheet_5.PDF]

Table S2. The mutations of S protein in HNU4-1 compared to HKU28

Site	Region	Mutation	Change of aa properties
5	S1	I5V	No
8	S1	T8L	(polar, none-charge) to (non-polar, none-charge)
9	S1	I9M	No
12	S1	A12V	No
13	S1	A13V	No
33	S1	Q33D	(polar, none-charge) to (polar, negative-charge)
36	S1	K36R	No
38	S1	T38S	No
40	S1	Q40D	(polar, none-charge) to (polar, negative-charge)
43	S1	L43I	No
45	S1	R45E	(polar, positive-charge) to (polar, negative-charge)
50	S1-NTD	N50R	(polar, none-charge) to (polar, positive-charge)
59	S1-NTD	D59A	(polar, negative-charge) to (non-polar, none-charge)
60	S1-NTD	S60N	No
62	S1-NTD	G62T	No
65	S1-NTD	S65Q	No
125	S1-NTD	S125P	(polar, none-charge) to (non-polar, none-charge)
162	S1-NTD	Q162L	(polar, none-charge) to (non-polar, none-charge)
163	S1-NTD	S163I	(polar, none-charge) to (non-polar, none-charge)
179	S1-NTD	S179E	(polar, none-charge) to (polar, negative-charge)
180	S1-NTD	T180K	(polar, none-charge) to (polar, positive-charge)
190	S1-NTD	L190F	No
207	S1-NTD	Q207K	(polar, none-charge) to (polar, positive-charge)
253	S1-NTD	A253R	(non-polar, none-charge) to (polar, positive-charge)
256	S1-NTD	N256T	No
258	S1-NTD	P258N	(non-polar, none-charge) to (polar, none-charge)
273	S1-NTD	F273L	No
314	S1	M314R	(non-polar, none-charge) to (polar, positive-charge)
332	S1-CTD	D332Y	(polar, negative-charge) to (polar, none-charge)
355	S1-CTD	Y355H	(polar, none-charge) to (polar, positive-charge)
382	S1-CTD	K382T	(polar, positive-charge) to (polar, none-charge)
419	S1-CTD	N419T	No
455	S1-CTD	K455Q	(polar, positive-charge) to (polar, none-charge)
467	S1	Y467H	(polar, none-charge) to (polar, positive-charge)
486	S1	R486K	No
564	S1	V564I	No
567	S1	D567E	No
570	S1	D570N	(polar, negative-charge) to (polar, none-charge)
571	S1	A571T	(non-polar, none-charge) to (polar, none-charge)

Table S2 (continued)

Site	Region	Mutation	Change of aa properties
683	S2	E683D	No
686	S2	K686E	(polar, positive-charge) to (polar, negative-charge)
692	S2	N692Y	No
694	S2	E694Q	(polar, negative-charge) to (polar, none-charge)
698	S2	-698G	(None, None) to (polar, none-charge)
699	S2	V699L	No
700	S2	N700D	(polar, none-charge) to (polar, negative-charge)
769	S2	K769T	(polar, positive-charge) to (polar, none-charge)
1012	S2	S1012N	No
1029	S2	I1029M	No
1124	S2	I1124V	No
1176	S2	G1176A	(polar, none-charge) to (non-polar, none-charge)
