# Supplementary material for: Characterization of Deltacoronavirus in Black-Headed Gulls (Chroicocephalus ridibundus) in South China Indicating Frequent Interspecies Transmission of the Virus in Birds
Source: Front Microbiol. 2022 May 12;13:895741. doi: 10.3389/fmicb.2022.895741 (PMC9133700; doi:10.3389/fmicb.2022.895741)
Supplement: Supplementary file 6 [file Data_Sheet_6.PDF]

Table S3. The taxonomy of host of deltacoronavirus.

Host name	Family	Order
Pygoscelis papua	*Spheniscidae*	*Sphenisciformes*
Nycticorax nycticorax	*Ardeidae*	*Pelecaniformes*
Arenaria interpres	*Scolopacidae*	*Charadriiformes*
Chroicocephalus ridibundus	*Laridae*	*Charadriiformes*
Columba livia	*Columbidae*	*Columbiformes*
Chlamydotis macqueenii	*Otididae*	*Gruiformes*
Falco peregrinus tundrius	*Falconidae*	*Falconiformes*
Gallinula chloropus	*Rallidae*	*Gruiformes*
Coturnix coturnix	*Phasianidae*	*Galliformes*
Pica pica	*Corvidae*	*Passeriformes*
Zosterops japonicus	*Zosteropidae*	*Passeriformes*
Pycnonotus sinensis	*Pycnonotidae*	*Passeriformes*
Montifringilla taczanowskii	*Passeridae*	*Passeriformes*
Passer montanus	*Passeridae*	*Passeriformes*
Lonchura striata	*Estrildidae*	*Passeriformes*
Turdus hortulorum	*Turdidae*	*Passeriformes*
Copsychus saularis voucher	*Muscicapidae*	*Passeriformes*
Sus scrofa	*Suidae*	*Artiodactyla*
Prionailurus bengalensis	*Felidae*	*Carnivora*
Mareca penelope	*Anatidae*	*Anseriformes*
